# Validity of Clinical Assessment Using Clinical Symptoms and C-Reactive Protein for Therapeutic Response in Pyogenic Vertebral Osteomyelitis: Analysis Based on ^18^F-FDG-PET

**DOI:** 10.3390/medicina57080809

**Published:** 2021-08-06

**Authors:** Ikchan Jeon, Dongwoo Yu, Eunjung Kong

**Affiliations:** 1 Department of Neurosurgery, Yeungnam University College of Medicine, Daegu 42415, Korea; icarus0810@hanmail.net; 2 Department of Nuclear Medicine, Yeungnam University College of Medicine, Daegu 42415, Korea; kongej@yu.ac.kr

**Keywords:** pyogenic, vertebral osteomyelitis, therapeutic response, clinical assessment, positron emission tomography, false positive

## Abstract

*Backgroundand objectives:* The clinical assessment of therapeutic response in pyogenic vertebral osteomyelitis (PVO) has been usually performed based on the changes of clinical symptoms and blood inflammatory markers. Recently, ^18^F-fluorodeoxyglucose positron emission tomography (^18^F-FDG-PET) has emerged as an alternative independent method. We analyzed the validity of the clinical assessment for detecting residual PVO based on ^18^F-FDG-PET. *Materials and Methods:* This study was conducted with 53 patients confirmed as lumbar PVO under retrospective design. All patients underwent clinical assessment using clinical symptoms and C-reactive protein (CRP) for therapeutic response after parenteral antibiotic therapy, which led to the decision of placement in the uncontrolled (group UC) or controlled (group C) group. The validity of clinical assessment was analyzed based on the cut-off values of FDG uptake for detecting residual PVO as references, which are already established in the previous literature. *Results*: The mean duration of parenteral antibiotic therapy and recurrence rate were 42.19 ± 15.84 (21–89) days and 9.4% (5/53), respectively. ^18^F-FDG-PETs were performed at 80 rounds of clinical assessment on 37.40 ± 13.15 (21–83) days of parenteral antibiotic therapy and divided: 31 into group UC and 49 into group C, according to the decisions of clinical assessment. Based on the cut-off values of FDG uptake, clinical assessment showed 48.4–58.1% of false positive for residual PVO in group UC. However, ^18^F-FDG-PET showed 8.2% (4/49) of false negative for residual PVO in group C, which led to recurrences. *Conclusions:* Clinical assessment using clinical symptoms and CRP for evaluating therapeutic response in PVO is still a useful method in terms of similar recurrence rate compared to ^18^F-FDG-PET. However, the high rate of false positive for residual PVO can prolong the use of unnecessary antibiotics and overall treatment period.

## 1. Introduction

Spine infection is an infectious disease of the vertebrae and adjacent structures presented as vertebral osteomyelitis and can develop from pyogenic, tuberculous, and other causes [1]. Among spine infection types, pyogenic vertebral osteomyelitis (PVO) is most common. In the United States, the annual incidence of hospitalization due to PVO is 5.4 per 100,000 persons in 2013 [2]. PVO shows non-specific symptoms and may not necessarily present with fever [3,4]. Usually, PVO would have already progressed with spondylodiscitis and abscess of epidural or paravertebral structures before diagnosis [5]. Approximately half of PVO cases are still treated with empirical antibiotics because of culture-negative for causative microorganism despite several culturing attempts [6]. PVO usually requires conservative treatment with long-term antibiotics; recently, Infectious Diseases Society of America recommended six weeks of antibiotic therapy for PVO with parenteral or a highly bioavailable oral agent [3]. However, there are still no clear guidelines for treating PVO due to variability in causative microorganisms and antibiotic resistance among the regions [7].

The assessment of therapeutic response after antibiotic therapy is still mainly based on clinical symptoms and blood inflammatory markers, including erythrocyte sedimentation rate (ESR), C-reactive protein (CRP), and white blood cell (WBC) count. However, clinical symptoms, such as pain, usually vary among the patients and lack objectivity, and blood inflammatory markers are easily affected by other conditions. As a result, the clinical assessment using clinical symptoms and hematological inflammatory markers is still unclear to determine whether residual PVO remains and the timing of discontinuing antibiotic therapy [5]. Magnetic resonance imaging (MRI) is considered as the best method to show the anatomical resolution and diagnosis of PVO. However, it is difficult to distinguish residual PVO lesions from the post-treatment structural changes and damages caused by PVO. Moreover, even in the favorable treatment progress with clinical recovery, MRI can show even more deteriorated condition compared to that of previous imaging findings because restoration of structural damages caused by PVO takes several months [8,9].

In the assessment of therapeutic response in spine infection, the attempts to apply ^18^F-fluorodeoxyglucose positron emission tomography (^18^F-FDG-PET) have been going on for 10 years, and detailed research results have been reported [10,11]. ^18^F-FDG-PET is considered to be less affected by other conditions than blood inflammatory markers [12]. The change of the intensity of FDG uptake presented as a maximum standardized uptake value (SUV_max_) between before and after antibiotic therapy was considered a useful method for therapeutic response [12,13]. Recently, Jeon et al. [14] reported that ^18^F-FDG-PET had higher diagnostic accuracy than CRP and MRI for detecting residual PVO using the intensity of FDG uptake. Furthermore, they emphasized the increase of diagnostic accuracy for detecting residual PVO using ^18^F-FDG-PET and CRP together. In addition, the clinical and radiological characteristics according to anatomical location of major structural damage were analyzed in patients with cured PVO, which was expected to be very helpful for determining therapeutic response in clinical field [15].

In this study, we analyzed the validity of clinical assessment for therapeutic response in PVO based on the intensity of FDG uptake on ^18^F-FDG-PET considering the advantages of ^18^F-FDG-PET and the limitations of clinical assessment using clinical symptoms and CRP.

## 2. Patients and Methods

### 2.1. Patients

This study was conducted with prospectively collected clinical and radiological data of 80 patients (49 men and 31 women) from February 2017 to September 2020 under retrospective design in a single institution. The patients presented with clinical symptoms of fever, back pain, or neurological signs (radiculopathy, weakness, or bowel/bladder symptoms) with specific findings of contiguous single lumbar PVO on MRI with/without causative bacterial cultures in PVO lesion or >2 sets of blood cultures [14,16]. The exclusion criteria is as follows: tuberculous vertebral osteomyelitis, PVO lesion containing instrumentation or bone cement, bone infection other than spine, trauma, tumor, pregnancy, under the severe medical problems, or were <20 years. All patients who participated in this study provided voluntary written informed consent to perform an additional simultaneous ^18^F-FDG-PET/MRI in each clinical assessment, and all clinical and radiological data were obtained and analyzed under the approval of the institutional review board (Yeungnam University Hospital, 2016-12-019-013, and 22 December 2016).

### 2.2. Clinical Assessment of Therapeutic Response after Antibiotic Therapy

**Figure 1:**
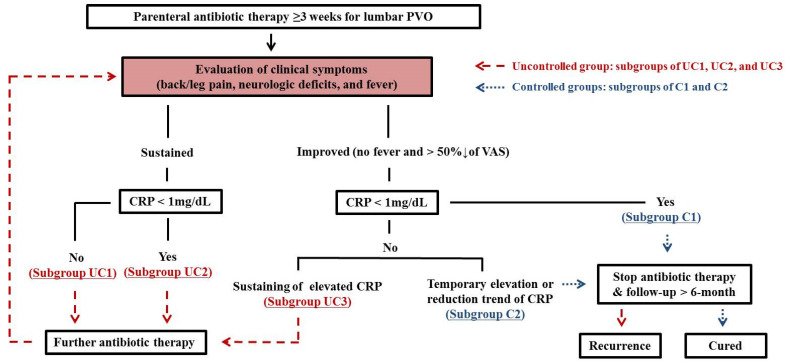
The algorithm of clinical assessment of therapeutic response (PVO-flowchart). Finally, 53 patients underwent 80 rounds of clinical assessment of therapeutic response based on a PVO flowchart during antibiotic therapy. The decisions of therapeutic response consisted of 36 Uncontrolled and 44 Controlled. Simultaneous ^18^F-FDG-PET/MRIs were performed at each clinical assessment (1.51 times/patient). PVO, pyogenic vertebral osteomyelitis; CRP, C-reactive protein; VAS, visual analog scale; ^18^F-FDG-PET/MRI, ^18^F-fluorodeoxyglucose positron emission tomography/magnetic resonance imaging.

All patients who participated in this study underwent clinical assessments for therapeutic response based on PVO-flowchart (Figure 1) after at least three weeks of parenteral antibiotics. The clinical assessments were then accordingly decided as belonging to the Uncontrolled (group UC) or Controlled (group C) group. PVO flowchart includes clinical symptoms and CRP level (normal range of <0.5 mg/dL in our institute). Visual analogue scale (VAS) was used to measure back/leg pain intensity, ranging from 0 (no pain) to 10 (maximum pain). Improvement was defined as pain alleviation by >50% according to VAS. Simultaneous ^18^F-FDG-PET/MRI (Biograph mMR; Siemens Healthcare, Erlangen, Germany) on lumbar spine was performed at each clinical assessment. The choice of effective parenteral antibiotics was performed under the consultation of infectious disease physicians. When a patient was classified into group C on clinical assessment, parenteral antibiotic therapy was discontinued. All patients underwent follow-up period of ≥6 months after discontinuing parenteral antibiotics; then, they were confirmed as cured if there was no recurrence. The subgroups “Cured” and “Recurrence” are defined below.

#### 2.2.1. Uncontrolled (Group UC)

- Subgroup UC1: sustained back/leg pain and/or aggravation of neurological deficits (weakness or bowel/bladder symptoms) with/without fever and persistent CRP ≥ 1 mg/dL
- Subgroup UC2: the same clinical criteria as subgroup UC1 with CRP < 1 mg/dL
- Subgroup UC3: persistent CRP ≥ 1 mg/dL even with improved back/leg pain and neurological deficits with no fever

#### 2.2.2. Controlled (Group C)

- Subgroup C1: improved back/leg pain and neurological deficits with no fever as well as CRP < 1 mg/dL
- Subgroup C2: the same clinical criteria as subgroup C1 with CRP ≥ 1 mg/dL but with an overall decreasing trend or temporary elevation of CRP

#### 2.2.3. Cured and Recurrence

- Cured: defined as absence of fever, improved clinical symptoms, and a sustained trend of normalized CRP after follow-up period of ≥6 Months.
- Recurrence: re-elevation of CRP ≥1 mg/dL, recurred back/leg pain and/or neurological deficits with/without fever and development of new or aggravation of PVO on MRI within follow-up period.

### 2.3. Analysis of PVO-Flowchart Using the Cut-Off Values of FDG Uptake for Detecting Residual PVO

The differences of intensity of FDG uptake between the groups UC and C as well as according to the subgroups were analyzed retrospectively. We used the cut-off values of FDG-uptake parameters for detecting residual PVO presented by Jeon et al. [14], which include the maximum standardized uptake value of PVO lesion (PvoSUV_max_, cut-off value of 6.44), difference between PvoSUV_max_ and SUV_max_ of normal vertebra (RefSUV_max_) (Δ PvoSUV_max_–RefSUV_max_, cut-off value of 4.50), and difference between PvoSUV_max_ and the mean standardized uptake value of normal vertebra (RefSUV_mean_) (ΔPvoSUV_max_–RefSUV_mean_, cut-off value of 4.84). The normal vertebra located two segments away from the PVO lesion were used as reference. When FDG uptake parameters are above each cut-off value, it is defined as having a residual PVO. Based on the confirmed cut-off values of FDG-uptake parameters for detecting residual PVO, false-positive cases in group UC and false-negative cases in group C on the PVO flowchart were analyzed.

### 2.4. PET/MRI Data Acquisition

Simultaneous ^18^F-FDG-PET/MRIs were taken centered on the lumbar spine. All patients fasted for at least six hours to maintain blood glucose level of <8.9 mmol/L before injecting FDG (3.7 MBq/kg). The acquisition of simultaneous ^18^F-FDG-PET/MRI initiated at 60 min after FDG injection and lumbar area was scanned in one–two bed positions using the approved surface coil. ^18^F-FDG-PET data acquisition occurred over 20 min, and MRI data were simultaneously obtained according to the protocol of Jeon et al. [14]. A 3-dimensional ordered subsets expectation maximization iterative reconstruction (OSEM-IR) algorithm was applied with three iterations and 21 subsets for ^18^F-FDG-PET data using 172 × 172 matrix.

### 2.5. Statistical Analysis

Student’s *t*-test and Mann–Whitney U test were used to compare the two population means for parametric continuous variables and non-parametric continuous variables, respectively. Statistical analyses were conducted with SPSS version 25.0 software (SPSS Inc., Chicago, IL, USA), and the probability value (*p*-value) of <0.05 was considered statistically significant.

## 3. Results

### 3.1. Patients

Among the 80 patients, 27 patients were finally excluded for the following reasons: lost to follow-up or participation withdrawal (*n* = 6), bone infection other than spine (*n* = 1), PVO lesion containing instrumentation or bone cement (*n* = 5), paraspinal or back muscle abscess without spondylodiscitis (*n* = 5), and misdiagnosis (*n* = 10; four of tuberculous spinal infection, two of trauma, two of ankylosing spondylitis, and two of degenerative change). The final analyses were performed on the remaining 53 patients (32 men and 21 women) with a mean age of 67.00 ± 11.41 (31–85) years. Procedure-related occurrences, including injection, acupuncture, or operation, were the main cause of PVO (32/53, 60.4%). There were five cases of recurrence (9.4%, 5/53) during the follow-up period of more than 6 months. Detailed data are described in Table 1.

**Figure 2:**
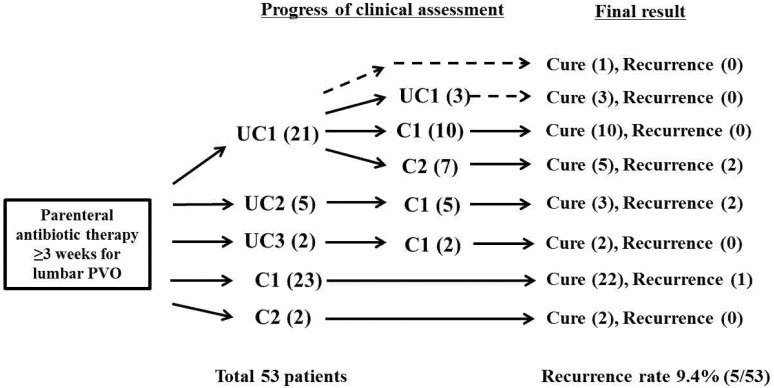
The overall clinical assessments and its decisions for therapeutic response during parenteral antibiotic therapy. PVO, pyogenic vertebral osteomyelitis; ^18^F-FDG-PET/MRI, ^18^F-fluorodeoxyglucose positron emission tomography/magnetic resonance imaging.

Fifty-three patients underwent a total of 80 rounds of clinical assessment based on the PVO flowchart. The decisions of therapeutic response consisted of 31 in group UC and 49 in group C, which were based on the clinical assessments performed on 33.19 ± 10.68 (21–57) and 40.06 ± 13.95 (21–83) days of parenteral antibiotic therapy, respectively (*p* = 0.022). Simultaneous ^18^F-FDG-PET/MRIs were performed at each clinical assessment (once or twice per patient). The overall clinical assessments and its decisions for therapeutic response during parenteral antibiotic therapy are summarized in Figure 2.

### 3.2. Causative Bacteriae and Antibiotics

The rate of causative bacterial identification was 50.9% (27/53) with blood or tissue culture, and the most common causative bacteria was methicillin-sensitive *Staphylococcus aureus* (MSSA). The mean duration of parenteral antibiotic therapy was 42.19 ± 15.84 (21–89) days. Five male patients (one of methicillin-resistant *S. epidermidis* and four of culture-negative patients) showed recurrence after the discontinuation of parenteral antibiotic therapy. There was no statistical difference in the duration of parenteral antibiotic therapy based on bacterial cultures (43.81 ± 19.85 (21–89) days of culture positive and 40.50 ± 10.33 (23–61) days of culture negative, *p* = 0.448). Details are presented in Table 2.

### 3.3. Overall Clinical Assessment and Its Decision during Antibiotic Therapy

Fifty-three patients underwent total 80 rounds of clinical assessment based on PVO-flowchart with simultaneous ^18^F-FDG-PET/MRI during parenteral antibiotic therapy. The decisions were divided into subgroups UC1, UC2, and UC3 of the group UC and subgroups C1 and C2 of group C. Parenteral antibiotic therapy was discontinued in 25 patients with the decision of subgroups C1 (*n* = 23) and C2 (*n* = 2), while the remaining 28 patients underwent additional clinical assessments of therapeutic response during parenteral antibiotic therapy. Four patients of subgroup UC1 were cured after further antibiotic therapy and clinical assessment without performing simultaneous ^18^F-FDG-PET/MRI. Five recurrences were identified in three of subgroup C1 and two of subgroup C2. Details are presented in Figure 2.

Fifty-three patients underwent clinical assessment based on PVO flowchart during parenteral antibiotic therapy. Simultaneous ^18^F-FDG-PET/MRIs were performed in 80 rounds of clinical assessments. The decisions were divided into the subgroups UC1, UC2, and UC3 of Uncontrolled (group UC) and the subgroups C1 and C2 of Controlled (group C). Parenteral antibiotic therapy was discontinued in 25 patients with the decisions of C1 (*n* = 23) and C2 (*n* = 2), while the remaining 28 patients underwent additional clinical assessments of therapeutic response during parenteral antibiotic therapy. Four patients of subgroup UC1 were cured after further parenteral antibiotic therapy without dividing into group C based on ^18^F-FDG-PET/MRI (broken arrows). There was 9.4% of recurrence (5/53).

### 3.4. Validity of PVO Flowchart Based on ^18^F-FDG-PET

False-positive rates for residual PVO of PVO flowchart are as shown below. When the ≥ 6.44 of PvoSUV_max_ cut-off value for residual PVO was used, 48.4% (15/31) of group UC was identified as no residual PVO. When the ≥4.50 of Δ PvoSUV_max_–RefSUV_max_ cut-off value was used, 58.1% (18/31) of group UC was identified as no residual PVO. When the ≥4.84 of Δ PvoSUV_max_–RefSUV_mean_ cut-off value was used, 58.1% (18/31) of group UC was identified as no residual PVO.

False-negative rate for residual PVO confirmed as recurrence of PVO flowchart was 10.2% (5/49) based on the analysis in group C. However, overall false-positive rate based on the all of 53 participants was 9.4% (5/53), as aforementioned. In false-negative rate of ^18^F-FDG-PET, when the cut-off values of PvoSUV_max_, Δ PvoSUV_max_–RefSUV_max_, and Δ PvoSUV_max_–RefSUV_mean_ are used, false-negative rates were 8.2% (4/49) in all FDG-uptake parameters. Details are presented in Table 3.

### 3.5. Differences of CRP and FDG Uptake between Subgroups

In CRP, subgroups UC1 (*n* = 24), UC2 (*n* = 5), UC3 (*n* = 2), R (*n* = 5, confirmed as recurrence), C1 (*n* = 37), and C2 (*n* = 7) showed 3.63 ± 2.82 (0.50–11.47), 0.46 ± 0.34 (0.10–1.02), 3.51 ± 1.39 (2.54–4.51), 1.14 ± 0.97 (0.02–2.27), 0.71 ± 1.05 (0.02–6.30), and 2.95 ± 1.89 (1.07–5.93), respectively. In PvoSUV_max_, subgroups UC1, UC2, UC3, R, C1, and C2 showed 7.22 ± 2.56 (2.80–14.19), 6.34 ± 1.78 (4.47–9.08), 6.08 ± 0.85 (5.48–6.68), 5.26 ± 2.39 (3.52–9.35), 4.98 ± 2.50 (2.10–16.11), and 5.03 ± 1.79 (2.31–6.84), respectively. Δ PvoSUV_max_–RefSUV_max_ and Δ PvoSUV_max_–RefSUV_mean_ showed similar trend between subgroups compared to PvoSUV_max_; detailed data are presented in Table 4.

## 4. Discussion

It is still difficult to determine when to discontinue antibiotic therapy due to inconsistencies in clinical symptoms and blood inflammatory markers in PVO. Thus, there are no clear guidelines for determining therapeutic response. Generally, CRP is more strongly associated with clinical symptoms compared to ESR and WBC count, which decreases quickly in the patients presenting with clinical improvement [17]. Especially, WBC count is proved to be less indicative of the diagnosis and treatment response and produces false negatives in cases of elderly or immunosuppressed patients [18,19]. Given these findings, we designed a PVO flowchart for the clinical assessment of therapeutic response using clinical symptoms and CRP level based on the literature and our clinical experiences. The proposed minimum duration of parenteral antibiotic therapy for PVO is 2–4 weeks [16,20,21]. In our study, the minimum required duration of parenteral antibiotic therapy for clinical assessment was determined to three weeks based on previous studies recommending short treatment duration [20,22]. Finally, clinical assessment of therapeutic response was performed based on clinical symptoms and CRP level after at least three weeks of parenteral antibiotic therapy. Recently, the efficacy of oral antibiotics as the first-line therapy has been reported; however, our study used parenteral antibiotics, as the evidence for the efficacy of oral antibiotics is still insufficient, and there are higher rates of antibiotic resistance. Furthermore, we considered that high-bioavailability oral antibiotics are not available under our national health-insurance system.

**Figure 3:**
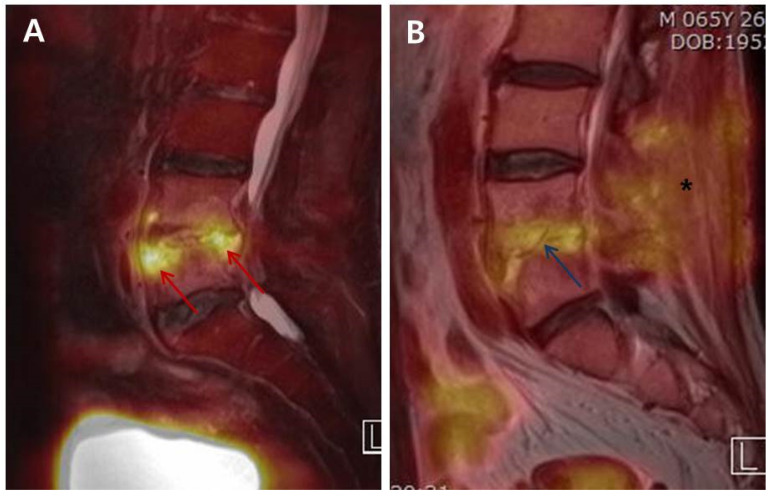
Differences in the intensity of FDG uptake between Uncontrolled (group UC) and Controlled (group C) simultaneous ^18^F-FDG-PET/MRIs. (**A**) The 60-year-old male patient diagnosed as PVO on L4–L5 was treated with empirical vancomycin and ciprobay under the no causative bacterial identification. The clinical assessment on the 36th day of parenteral antibiotic therapy showed sustained back pain, intermittent mild fever, and ESR/CRP of 57/7.726. ^18^F-FDG-PET/MRI of group UC revealed an increased FDG uptake (red arrow; SUV_max_ 9.26) on the anterior and posterior corners of upper L5 body and the disc of L4-L5. (**B**) The 65-year-old male patient diagnosed as postoperative PVO on L4–L5 was treated with nafcillin under the identification of MSSA. The patient showed alleviation in back pain, no fever, and ESR/CRP of 30/0.09 in the clinical assessment on the 35th day. The FDG uptake decreased overall and was limited to the disc and endplates (blue arrow; SUV_max_ 3.82) on ^18^F-FDG-PET/MRI of group C. There was also decrease in FDG uptake at posterior structures, including back muscle and fatty layer (asterisk). FDG, ^18^F-fluorodeoxyglucose; PVO, pyogenic vertebral osteomyelitis; MSSA, methicillin-sensitive *Staphylococcus aureus*; ESR, erythrocyte sedimentation rate (mm/h); CRP, C-reactive protein (mg/dL); ^18^F-FDG-PET/MRI, ^18^F-fluorodeoxyglucose positron emission tomography/magnetic resonance imaging; SUV_max_, maximum standardized uptake value of FDG.

Jeon et al. [14,15] investigated the change of FDG uptake according to the phases of osteomyelitis based on the pathophysiological features of osteomyelitis. The early phase of PVO is characterized by activated neutrophil accumulation, known as the respiratory burst, which uses greater amounts of glucose as the main energy source for chemotaxis and phagocytosis [23]. In the chronic phase, lymphocytes are the most predominant cell type, followed by plasma cells, histiocytes, and some polymorphonuclear leucocytes [24]. The transport of FDG across the cellular membrane is mediated by the glucose transporter of cell membrane, which is more abundantly found on the activated inflammatory cells [25]. Consequently, FDG uptake increases by the activated granulocytes in the acute phase. On the other hand, in the chronic or recovery phase, lymphocytes are the predominant inflammatory cells. In addition, there are formations of fibroses around the foci of inflammation and bone marrow, fatty changes, increased osteoblasts facilitating new bone formation, and dilated blood vessels [24]. The mechanical stress on the intervertebral disc and endplates depends on the patient’s activities in addition to the formation of fibrosis and granulation tissues during the recovery phase [15]; these can result in the sustained increase of FDG uptake at the intervertebral disc and endplates even after successful treatment compared to widely FDG uptake on uncontrolled cases (Figure 3).

In clinical assessment, we considered clinical symptoms, fever in particular, to be the most important factor in assessing therapeutic response. Interpretation of the CRP level was based on a reference threshold of 1 mg/dL. If clinical symptoms persisted, parenteral antibiotic therapy was continued regardless of the CRP level. Improvement in clinical symptoms with a CRP level of <1 mg/dL was the most significant finding that led to discontinuation of parenteral antibiotics. In cases showing a temporary increase or a persistent decreasing trend of the CRP level compared with initial measurement, even if the CRP level was ≥1 mg/L, parenteral antibiotic therapy was discontinued. In these cases, continued elevation of the CRP level was possibly due to mechanical stress on damaged tissues of intervertebral structure caused by the patient’s movement. Cured PVO showed various clinical features associated with the location of main structural damage of the PVO lesion [15]. The involvement of intervertebral structure as main structural damage was related to sustained back pain and elevation of CRP. On the contrary, vertebral body/paravertebral muscle showed favorable clinical features despite more advanced structural damages with higher FDG uptake. The mechanical stress exerted by the patient’s activities may affect the damaged intervertebral disc and endplates more than the vertebral body or paravertebral muscle, which is related to the sustained elevation of the CRP level and back pain even in cases with cured PVO.

We think that the PVO flowchart has proven a relatively favorable reliability, with a recurrence rate of 9.4% (5/53) for the assessment of therapeutic response in PVO. The recurrence rates reported in previous literature vary greatly. McHenry et al. [26] reported a recurrence rate of 14% in 253 patients; Park et al. [27] of 9.9% in 314 patients; and Kim et al. [7] of 6.6% in 151 patients. However, there are still insufficient data to guide the optimal duration of antibiotic therapy related to the recurrence of PVO [6,28,29]. Some studies have reported that less than 6–8 weeks of antibiotic therapy may be highly associated with a risk of recurrence [7,26,30,31]. Kim et al. [7] reported a lower recurrence rate of 6.6% with an average duration of over 100 days with total antibiotic therapy. Based on these results, we think that a sufficient duration of antibiotic therapy may be helpful in preventing recurrence. However, considering the adverse effects and complications associated with long-term antibiotic therapy, it is important to use a minimum period of antibiotics while increasing the success rate of treatment.

Clinical assessment using the PVO flowchart can be a reliable method for assessing therapeutic response considering the low recurrence rate. There were inconsistencies between clinical symptoms, CRP, and FDG uptake. In particular, sustained symptoms in subgroups UC1 and UC2 may lead to use of unnecessary antibiotics and re-assessment. There is a possibility of overestimation for residual PVO in group UC, which can be considered as false positive under the analysis using the cut-off values of FDG uptake for detecting residual PVO. The rates of false positive were 48.4% (15/31) in PvoSUV_max_, 58.1% (18/31) in Δ PvoSUV_max_–RefSUV_max_, and 58.1% (18/31) in Δ PvoSUV_max_–RefSUV_mean_, respectively. Considering that Jeon et al. [14] reported a diagnostic accuracy of about 85% for residual PVO based on FDG-uptake parameters, the real false-positive rate can be predicted at around 40–50% on clinical assessment using the PVO flowchart. However, unfortunately, it is very difficult to differentiate false positives in the clinical field due to the low rate of causative bacterial identification even by repeated culture tests for PVO lesion. Given these points, we can identify the limitation of the clinical assessment in shortening the duration of antibiotic therapy due to the high rate of false positive for residual PVO.

Although our study had tried a new attempt to evaluate the validity of clinical assessment using clinical symptoms and CRP level based on ^18^F-FDG-PET, there are several limitations. First, this study was conducted under a retrospective study design with a relatively small number of patients despite the use of prospectively collected data based on the designed PVO flowchart. Second, we used the FDG-uptake parameters with approximately 85% diagnostic accuracy for detecting residual PVO; it is not possible to confirm false positives accurately unlike false negatives that could be confirmed as a recurrence.

## 5. Conclusions

Clinical assessment using clinical symptoms and CRP level for therapeutic response in PVO is still a useful method in terms of recurrence rate. However, the possibility of high rate of false positive for residual PVO can prolong the use of unnecessary antibiotics and the overall treatment period. Further studies with a large number of patients and applying various modalities, such as ^18^F-FDG-PET, under the prospective design are required to obtain a more reliable assessment method for therapeutic response in PVO.

## Figures and Tables

**Table 1:** Demographic and clinical data of 53 patients.

Factors	Values
Age, years	67.00 ± 11.41 (31–85)
Sex	Male 32, Female 21
Extension of PVO, units	1.38 ± 0.53 (1–3)
Cause of PVO	
Spontaneous	21/53 (39.6%)
Procedure-related	32/53 (60.4%)
Injection or acupuncture	25/32 (78.1%)
Operation	7/32 (21.9%)
Comorbidity	
Diabetes mellitus	20/53 (37.7%)
Hypertension	27/53 (50.9%)
Hemodialysis	2/53 (3.8%)
Cerebrovascular disease	4/53 (7.5%)
Ischemic heart disease/Arrythmia/Heart failure (7/2/1)	10/53 (18.7%)
Asthma/Chronic obstructive lung disease (1/1)	2/53 (3.8%)
Previous cancer history	4/53 (7.5%)
Clinical symptoms	
Fever	30/53 (56.6%)
Back pain	51/53 (96.2%)
Neurologic deficits	
Radiculopathy	31/53 (58.5%)
Weakness	6/53 (11.3%)
Bowel/Bladder symptoms	1/53 (1.9%)
Recurrence	5/53 (9.4%)
Duration of follow-up, months	12.34 ± 7.16 (6–35)

PVO, pyogenic vertebral osteomyelitis.

**Table 2:** Characteristics of bacteriology and antibiotics.

Factors	Values	
Bacterial identification	27/53 (50.9%)	
Culture positive	27	
Gram-positive bacteria	27	
Staphylococcus aureus	14	
MSSA	9	
MRSA	5	
Coagulase-negative staphylococci	5	
MRSE	2	
Others	3	
Enterococcus spp.	3	
Streptococcus spp.	5	
Gram-negative bacteria	0	
Culture negative	26	
Duration of parenteral antibiotic therapy, days	42.19 ± 15.84 (21–89)	
Culture positive (27/53, 50.9%)	43.81 ± 19.85 (21–89)	*p* = 0.448
Culture negative (26/53, 49.1%)	40.50 ± 10.33 (23–61)	

MSSA, methicillin-sensitive *Staphylococcus aureus*; MRSA, methicillin-resistant *Staphylococcus aureus*; MRSE, methicillin-resistant *Staphylococcus epidermidis*; PVO, pyogenic vertebral osteomyelitis; *p* value of <0.05 was considered statistically significant.

**Table 3:** Validity of PVO flowchart based on ^18^F-FDG-PET.

		Decisions of PVO Flowchart		
		Group UC (*n* = 31)	Group C (*n* = 49)	
FDG-Uptake Parameters			Cured	Recurrence
PvoSUV_max_ (cut-off value 6.44)	<6.44	15 (48.4%) *	38 (77.6%)	4 (8.2%) ^a^
	≥6.44	16 (51.6%)	6 (12.2%)	1 (2.0%)
Δ PvoSUV_max_–RefSUV_max_ (cut-off value 4.50)	<4.50	18 (58.1%) *	39 (79.6%)	4 (8.2%) ^a^
	≥4.50	13 (41.9%)	5 (10.2%)	1 (2.0%)
Δ PvoSUV_max_–RefSUV_mean_ (cut-off value 4.84)	<4.84	18 (58.1%) *	40 (81.6%)	4 (8.2%) ^a^
	≥4.84	13 (41.9%)	4 (8.2%)	1 (2.0%)

^18^F-FDG-PET, ^18^F-fluorodeoxyglucose positron emission tomography; PVO, pyogenic vertebral osteomyelitis; SUV_max,_ maximum standardized uptake value of ^18^F-fluorodeoxyglucose; SUV_mean,_ mean standardized uptake value of ^18^F-fluorodeoxyglucose; PvoSUV_max_, SUV_max_ of PVO lesion; Δ PvoSUV_max_–RefSUV_max_, difference between PvoSUV_max_ and SUV_max_ of normal vertebra (reference); Δ PvoSUV_max_–RefSUV_mean_, difference between PvoSUV_max_ and SUV_mean_ of normal vertebra; * false-positive decisions of PVO flowchart for residual PVO based on the cut-off values of FDG-uptake parameters; ^a^ False negatives of FDG-uptake parameters for residual PVO confirmed as recurrence.

**Table 4:** Differences of CRP and FDG uptake between subgroups.

	UC1 (*n* = 24)	UC2 (*n* = 5)	UC3 (*n* = 2)	R (*n* = 5)	C1 (*n* = 37)	C2 (*n* = 7)
CRP (mg/dL)	3.63 ± 2.82 (0.50–11.47)	0.46 ± 0.34 (0.10–1.02)	3.51 ± 1.39 (2.54–4.51)	1.14 ± 0.97 (0.02–2.27)	0.71 ± 1.05 (0.02–6.30)	2.95 ± 1.89 (1.07–5.93)
PvoSUV_max_	7.22 ± 2.56 (2.80–14.19)	6.34 ± 1.78 (4.47–9.08)	6.08 ± 0.85 (5.48–6.68)	5.26 ± 2.39 (3.52–9.35)	4.98 ± 2.50 (2.10–16.11)	5.03 ± 1.79 (2.31–6.84)
Δ PvoSUV_max_–RefSUV_max_	5.11 ± 2.67 (0.51–12.75)	4.45 ± 1.86 (2.38–7.11)	3.83 ± 0.46 (3.50–4.15)	3.04 ± 2.31 (1.32–7.06)	3.15 ± 2.44 (0.00–14.36)	2.96 ± 1.98 (0.00–5.29)
Δ PvoSUV_max_–RefSUV_mean_	5.50 ± 2.63 (0.96–12.97)	4.81 ± 1.92 (2.72–7.62)	4.20 ± 0.33 (3.96–4.43)	4.81 ± 1.92 (2.72–7.62)	3.48 ± 2.46 (0.06–14.64)	3.32 ± 1.98 (0.37–5.55)

CRP, C-reactive protein (normal range of <0.5 mg/dL in our institute); FDG, ^18^F-fluorodeoxyglucose; PVO, pyogenic vertebral osteomyelitis; SUV_max,_ maximum standardized uptake value of ^18^F-fluorodeoxyglucose; SUV_mean,_ mean standardized uptake value of ^18^F-fluorodeoxyglucose; PvoSUV_max_, SUV_max_ of PVO lesion; Δ PvoSUV_max_–RefSUV_max_, difference between PvoSUV_max_ and SUV_max_ of normal vertebra (reference); Δ PvoSUV_max_–RefSUV_mean_, difference between PvoSUV_max_ and SUV_mean_ of normal vertebra.

## Data Availability

The datasets acquired and analyzed during the current study are available from the corresponding author on the reasonable request.
